# Seniors’ perspectives on care: a case study of the Alex Seniors health clinic, Calgary

**DOI:** 10.1186/s13104-015-1008-0

**Published:** 2015-02-25

**Authors:** Marta Shaw, Candace Rypien, Neil Drummond, Patricia Harasym, Lara Nixon

**Affiliations:** Department of Family Medicine, University of Calgary, Faculty of Medicine, Health Sciences Centre, Foothills Campus, 3330 Hospital Drive NW, Calgary, AB T2N 4 N1 Canada; Faculty of Social Work, University of Calgary, 2500 University Dr. N.W, Calgary, AB T2N 1 N4 Canada; Department of Pediatrics, Faculty of Medicine, University of Calgary, Faculty of Medicine, Health Sciences Centre, Foothills Campus, 3330 Hospital Drive NW, Calgary, AB T2N 4 N1 Canada; Department of Family Medicine, University of Alberta, 1702 College Plaza, Edmonton, AB T6G 2C8 Canada; Department of Family Medicine, 1702 College Plaza, Edmonton, AB T6G 2C8 Canada; Department of Communication and Culture, Faculty of Communications and Culture, University of Calgary, 2500 University Dr. N.W, Calgary, AB T2N 1 N4 Canada

**Keywords:** Healthcare perspectives, Health services, Primary care, Community health centres, Elderly

## Abstract

**Background:**

Primary care initiatives face an imperative to not only reduce barriers to care for their patients but also to uniquely accommodate the complex needs of at-risk patient populations. Patient-centered multidisciplinary care team models for primary care, like the Alex Seniors Clinic, are one approach for providing comprehensive care for marginalized seniors. The purpose of this qualitative study was to explore patient perspectives on the responsiveness of the Alex Seniors Clinic to their stated health needs.

**Results:**

Themes reflected participants’ perspectives on factors impacting their health needs as vulnerable seniors as well as on the measures that the Alex Seniors Clinic has taken to meet those needs. Factors impacting health included: the nature of their relationships to the physical environment in which they lived, the nature of the relationships they had to others in that environment, and independence and autonomy. Participants identified accessibility, respect and support, and advocacy as the ways in which the clinic was working to address those health needs.

**Conclusions:**

While respect and support, as well as advocacy, effectively addressed some patient needs, participants felt that accessibility problems continue to be health-related barriers for clinic patients. This may be due to the fact that issues of accessibility reflect larger community and social problems. Nevertheless, it is only through engaging the patient community for input on clinic approaches that an understanding can be gained of how closely a clinic’s care goals are currently aligning with patient perspectives of the care and services they receive.

## Background

The healthcare of seniors requires a specialized approach as this population is vulnerable and has complex needs involving a myriad of physical and psychosocial issues. Some senior populations are especially vulnerable to poor health due to poverty. In Canada, 4.8% of seniors live below the poverty line, with this number growing to 13.9% among seniors who are unattached. The number of low-income seniors is expected to grow as seniors will make up 23% of the population by 2050 [1].

Research has demonstrated a relationship between urban economic vulnerability and poor health in older adults [2]. Seniors living in poverty are susceptible to a number of complex health stressors including low health literacy, chronic medical conditions and limited access to healthcare [3]. A possible explanation is that older residents in disadvantaged areas have limited resources to help them cope with the stress of their environment and health conditions. Disadvantaged urban areas are often characterized by a lack of medical screening facilities, health clinics, and health promoting social organizations [4]. Residents in disadvantaged neighborhoods may be at risk for poor health due to delays in, or lack of treatment for, both acute and chronic conditions. They may also disproportionately turn to unhealthy coping behaviors, such as cigarette smoking [5]. Urban socioeconomic disadvantage is strongly related to older adults’ perceptions of their health status [6]. Perceptions of health are predictive of actual health and mortality; socioeconomic disadvantage, therefore, may contribute to poor health due to lack of adequate resources and diminished self-perceived health status [7]. Designing health services that are comprehensive and extend beyond just medical care can be an effective way to support the complex needs of these vulnerable senior populations [8].

### Models of primary care delivery

Much attention worldwide has been paid to the optimization of primary care. Given the impact of social determinants on health, community interventions that address these issues are a current priority [2,6]. This builds on a long-term shift away from single-physician run practices and towards community-based primary care teams [8]. One team-based approach in Canada particularly aimed at disadvantaged populations is the community-governed, non-profit Community Health Centre (CHC). The CHC approach is mandated by the guiding principles of the Canadian Association of Community Health Centres (CACHC): support of a single-tiered health system; strong emphasis on health promotion and prevention; involvement of community residents, providers, and funders in identifying the needs of the community and in delivering and evaluating programs; willingness to take social and political action to improve the social, economic and environmental determinants of health; and work to remove unequal access to health services [9].

CHCs have been providing access to relevant and needed services for vulnerable populations in Canada since the early 1960s and have increased in number to more than 300 since the 1980s, largely in Quebec and Ontario [10]. CHCs work in partnership with other agencies, such as social services, justice, and education, that are committed to interdisciplinary practice and collaboration while responding to patients’ needs; they are a “one stop-community based hub” for primary medical care, social services and community development [10]. Community orientation (defined as care providers’ involvement and understanding of community’s needs) is significantly higher in CHCs compared with fee-for-service primary care models [11]. In addition, the inter-sectoral approaches used by CHCs have been shown to reduce health disparities [12]. Creating a patient-centered community health center in urban socioeconomically disadvantaged neighbourhoods is often a key strategy to assist vulnerable seniors with complex health needs. A key aspect of optimizing patient-centered care is having an understanding of patients’ perceived needs and their perceptions on how these needs are being met [13]. Good patient experience has a well-documented association with health care quality, including improved engagement with clinician instructions and improved clinical outcomes [14]. Knowledge of patient experiences is also a crucial step to understanding and improving care delivery.

### Study aims

The aim of this study was to explore the responsiveness of one CHC to the health needs of its senior patient population. We sought to answer the following research questions: what are vulnerable seniors’ perspectives on the factors that impact their health, and how do vulnerable seniors perceive the Alex Seniors Clinic’s attempts to address their complex health needs?

## Methods

### Participants and setting

The Alex Seniors Clinic began in the early 1970s due to an identified lack of coordinated care dedicated to the service of seniors living in poverty in the East Village and surrounding downtown areas of Calgary. It developed as a sub-division of the larger “Alexandra Community Health Centre” (‘the Alex Seniors Clinic’), which provides services for patients with multisystem complex healthcare needs, complicated by low income, limited education, a lack of social support and employment, as well as sub-optimal physical environments and health behaviors [15]. The Alex Seniors Clinic has systems in place to provide transportation and support when attending off-site appointments, as well as satellite clinics to better serve particularly vulnerable neighbourhoods. In addition, visiting specialists and home visits from nurses and physicians are provided for patients with major mobility limitations. Housing and food security programs at the Alex Seniors Clinic support these basic needs. Patients who are at-risk for forgetting appointments are given telephone and written reminders. In addition, measures are taken to help support and retain patients who exhibit disruptive behavior. Patients are supported independent of their health choices.

Within this context, the Alex Seniors Clinic staff includes family physicians and visiting medical specialists, a nurse practitioner, licensed practical nurses, nurse specialists (e.g. diabetes and chronic obstructive pulmonary disease educators), a community resource expert, pharmacist, recreation therapist, and massage therapist. The clinic has adopted a patient-centered multidisciplinary approach in an effort to best accommodate its urban, financially marginalized senior clientele. The clinic seeks to address issues related to self care through a number of programs, including satellite support care for substance abuse sufferers and complimentary health services (footcare, dietician, massage, psychology and recreation therapy).

All participants recruited to the study conformed to the 2005 eligibility criteria for patients of the clinic (age ≥ 55, income at or below Alberta poverty line, multi-morbidity, and living within a designated geographical area to facilitate access to clinic resources). Participants consisted of 12 men and 18 women with an age range of 62 to 95. All participants were Caucasian. Demographic characteristics of the sample were obtained from the clinic’s electronic medical records (Table 1).

**Table 1:** **Characteristics of the participant sample**

**Age**	**#**	**Number of health professionals Seen regularly at Alex Seniors Clinic**	**#**
<70	10	One	2
71-80	15	Two	14
81-90	4	Three	7
>90	1	Four	6
Gender		> Four	1
Male	12	Current number of medications	
Female	18	0-5	4
Housing		6-10	14
Subsidized next to clinic	9	11-15	9
Other subsidized	8	>15	3
Independent	5	Length of time at Alex Seniors Clinic	
With Family	2	<5 years	16
Other	6	>5 years	14
Education			
Some high school	17		
Completed high school	11		
University/college degree	2		
Driver’s License			
No current license	20		
Current License	7		
Data Unavailable	3		

### Procedure

An exploratory qualitative case study of the clinic was undertaken using semi-structured interviews with patients. This type of design was undertaken because it is best suited to studies in which the researchers want to answer questions pertaining to ‘how’. In this case, the researchers were interested in exploring how the clinic responds to patient needs. In addition, this type of design complements studies where the boundaries between context and phenomenon are blurred. In this case, it is not possible to fully separate the socioeconomic and geographic context of participants from their health needs [16]. The semi-structured interview allows the participant to determine the direction of the interview within the parameters of the topic area. It further enables participants to engage in deep reflection and individual exploration, ensuring richness in data content [17]. A convenience sample of 30 patients was interviewed by one of the researchers (CR) while they attended appointments at the clinic between January and May 2011. Given that transportation can be a major barrier for patients living in poverty, we judged that coupling participation in the study to a pre-existing clinic appointment would facilitate recruitment and increase heterogeneity in the sample. Thirty participants were selected, a number considered to be at the high end of suggested ranges (20–30) for qualitative research [18]. Patients were recruited to the study by the clinic’s reception staff. The purpose of the study and nature of participation was explained to all potential participants. Staff also communicated that participation was voluntary and participants could elect to end their participation at any time, without any repercussions. Patients were then asked to consent to participate and those who did were interviewed on-site, typically for 60 minutes (range 30–85 minutes), in a private room by CR, who was a senior medical student with previous master’s level research experience. An interview guide based on key known determinants of health (i.e. financial difficulty, housing, social support, food security, healthcare access, etc.) was used to explore seniors’ understanding of their own health, the services offered to them at the clinic and the processes by which the Alex made those services accessible to them (Table 2). All interviews were anonymized in order to remove all identifying information and to protect the confidentiality of participants. The study was approved by the Conjoint Health Research Ethics Board (CHREB) at the University of Calgary.

**Table 2:** **Semi-structured interview guide**

**Semi-structured interview questions**	
1. How long have you been coming to the Alex community Center?	6. Other then your medical health, what other things make it difficult for you to stay healthy?
Why did you start coming?	- Access to healthy Food?
Who do you see?	- Ease of getting around the city?
How often do you come?	- Safety in your community?
2. What did you want out of your visit today?	- Financial worries?
3. Have you had particularly good experiences at the Alex and can you tell me about them?	- Social contacts?
4. Have you had disappointing experiences at the Alex and can you tell me about them? If there were 2 things you could do to improve the Alex, what would they be?	- Housing?
5. There are several different types of health. Can you tell me what it means to you:	- Winter?
- to be physically healthy?	7. Do you have suggestion on how to improve the Alex?
- to be emotionally healthy?	8. Has the clinic helped you?
- to be spiritually healthy?	
- to live in a healthy environment?	
- to be socially content?	

### Data analysis

Interviews were audio-recorded, transcribed verbatim by a member of the research team, who was a psychology student with previous transcriptionist experience. Identifying information was removed to maintain anonymity. The transcribed interviews were read by the research team, consisting of a family physician (LN), pharmacist (PH), psychology student (MS), and medical student (CR). One of the researchers (LN) occupied a dual role as researcher and clinician. This researcher was not aware of the identities of the participants and while involved in the analysis, was only one voice in a consensus-based discussion. This researcher’s important role was to provide context for the setting and culture at the Alex Seniors Clinic. MS and CR used line-by-line coding to identify all aspects of the data. Using constant comparative methodology [18] initial codes were categorized on the basis of the first 15 interviews and CR’s field observations. These categories were agreed on by consensus following discussion among the researchers who met several times to iteratively review all of the transcripts. All interview transcripts were then re-read by CR and MS, who completed the coding. The codes were then entered into HYPEResearch [19] and subsequently organized inductively by the group into emerging themes, making sure to include all disconfirming cases into the emerging thematic framework. Quotes that best represent each theme were selected for inclusion in the final manuscript. An audit trail of analysis meetings amongst the researchers was kept and notes were reviewed as codes, themes and their definitions were developed. Key input was provided by ND, an epidemiologist who acted as peer reviewer [20]. To further enhance trustworthiness, once the framework was developed, it was revised to ensure that it accommodated disconfirming data on the basis of further examination of the interviews, constant comparative analysis, and consensus discussion.

## Results

**Figure 1:**
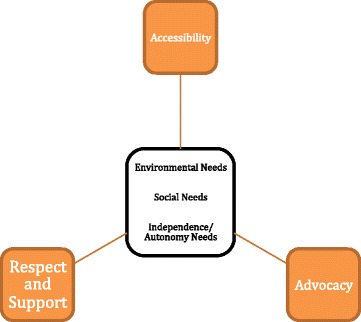
**Factors impacting health needs and ways in which Alex Seniors Clinic is responsive to stated needs.**

The themes that emerged from the data reflected participants’ perspectives on factors impacting their health needs as vulnerable seniors, as well as on the measures that the Alex Seniors Clinic has taken to meet those needs. Factors impacting health identified by participants included: the nature of the relationships respondents perceived themselves having to the physical environment within which they lived, the nature of the relationships they had to others in that environment, and independence and autonomy. Participants identified accessibility, respect and support, and advocacy as the ways in which the Alex Seniors Clinic was working to address those health needs (Figure 1). Each perceived health need, and the Clinic’s perceived method of response to it, is discussed below.

### Relationship to the environment

Aging was frequently described as being associated with a sense of increasing physical limitation dictated by struggles in participants’ interaction with their environment. Sense of security and feeling safe at home was a significant factor affecting health related to stress and anxiety. Participants discussed the stress of having to move to subsidized housing once they could no longer work. The lack of safety in their neighborhoods was a significant health concern associated with aging.

‘You have to worry about someone smashing the door down or a lot of drinking and arguing because, I mean, especially when you are a senior. Sure, I can handle myself but you don’t want to go to bed at night wondering if someone will bring your door down’.

In addition to personal safety, other environmental barriers included difficulty maneuvering through the public transportation system and physical dangers (e.g. icy sidewalks, snow banks, and streets under construction). Geographic accessibility to healthcare professionals and clinics emerged as a key environmental factor that appeared to impact the health of participants. Participants were wary of being outdoors in the winter due to increased risk of falls. Some expressed security concerns related to their place of residence and the community at large.

#### Accessibility

In addition to environmental factors, geographic accessibility factors at the clinic also emerged as a source of distress for patients, indicating an area of possible unmet needs.

‘I find it appalling…that I can’t come here if I live downtown.’

### Respect and support

Respondents acknowledged that the Alex Bus was useful for accessing the most vulnerable and isolated in the community but it was nevertheless felt that those who are hardest to reach are in greater need of additional supports, especially mental health staff.

‘I know the Alex bus is here but I don’t think they deal with mental issues, they are basically dealing to the street people which is a good thing but they can’t take on those issues. There should be more mental health workers in the system because as people get older, they really are scared of dying, eh. Somebody has to explain to them, it’s not really dying, it’s crossing over. Also, people are embarrassed of their mental issues- they don’t want to talk about their problems.’

### Advocacy

Participants noted that the clinic staff exemplified dedication to patients by advocating for those who have serious housing needs. One participant detailed his appreciation for the advocacy of a nurse practitioner at the Alex Seniors Clinic who was able to secure him subsidized housing when his former living arrangements had become unsafe.

‘She went to bat and she got me into Murdoch Manor right across the street. Normally, it’s a two-year waiting list and she got me in there in two months. She just fought and fought and got me in there! Who else can do that?’

### Relationships with others

Participants’ definition of psychosocial fulfillment was diverse but contentment in social relationships was identified as a factor impacting sense of health. While social needs varied widely among participants, the ability to choose the relationships they were in was of paramount importance. For some participants, strong interpersonal relationships contributed to a sense of social contentment:

‘I have a wonderful family. I just can’t imagine not having a family. They don’t all live here but being able to have that contact with them, that to me is so important. And when people don’t have anyone around, I can imagine that they just don’t want to live sometimes- I think that’s what happens. Sometimes, they lose the will to live when they’ve got no one at all.’

For other participants, a strong relationship with church or religious community was important:

‘The spiritual health is very important to me and friendship with others, that’s really important too. We all like being healthy but it doesn’t always work out that way.’

Others expressed the importance of volunteer work and the role this played on their outlook:

‘I like to mix with people. Like my volunteering is with people and that is very important to me. Not that I want people in my apartment all the time but I love having contact with people and connecting with them so doing my volunteering is enough for me.’

Some participants shared their preference for spending time alone and not needing the company of others to feel socially satisfied. These participants emphasized the importance of being in control of their social interactions.

‘I have always been kind of a loner. <I’m > socially content, I am just content to watch my sports on TV.’

Participants found different ways to relate and interact with their peers and health care professionals; they expressed varying degrees to which they desired social support versus independence. Still, patients who felt in control of their relationships expressed contentment.

### Accessibility

Some participants reported having difficulty accessing information on community services and programming. One participant indicated that she felt volunteering would be beneficial for her mental health but the Alex Seniors Clinic was not able to help her find a suitable position in the community.

‘I worked since I was 16 years old and I find it very difficult to be at home. I can’t stand it. It’s just not good for me mentally. I like to be out and about and socializing. I did come here once to speak to the lady about volunteering but um I kinda, well at the time, there wasn’t that much to offer…’

### Respect and support

Participants’ expressed satisfaction with interactions at the Alex Seniors Clinic was often related to how valued they felt by staff.

‘You always feel like a human being and they treat you with the utmost respect and dignity. I really can’t say enough about them. From the moment you come in the front door, until you leave. Makes you feel important-that they care.’

Another participant reflected on the personalized care she receives at the clinic, sharing that she had been asked to come to a medical appointment because her physician wanted to put her at ease regarding an upcoming procedure.

‘Just cause of my surgery coming up on January 31, which is Monday. So Dr. X just wanted to touch base today to make sure like I don’t have any questions. Let me know it’s gonna be ok. That kind of thing.’

### Advocacy

A number of participants commented on the high prevalence of social isolation among seniors living in subsidized housing in the East Village. They indicated that there is still work to be done for a clinic like the Alex Seniors Clinic to improve access and provide needed services to all members of the community.

‘You have to get out because that affects your health. As we were talking about, these isolated seniors, they can’t get out and get their groceries and that’s a huge thing. They are not getting their food so I don’t know what they do- that affects their health.’

### Independence and autonomy

Participants’ ability to take part in the physical world around them was a key factor that influenced their perceived health. The importance of maintaining one’s activities of daily living was emphasized, as was the ability to make autonomous decisions (e.g. medical, financial). Participants’ acceptance of their physical limitations was often paired with a focus on maintaining independence to the best of their ability.

‘You gotta have some responsibility for your own welfare. Everybody in the world can’t do it for you. You have got to give yourself a hundred percent effort in making sure that if you stay healthy…’

Participants with the most positive outlook were not necessarily the healthiest but rather expressed an understanding and acceptance of aging.

‘Like, nobody likes to go to the doctor’s office, let’s be honest, it’s an inconvenience, but it is a necessity and it is part of life when you get old and you need help from the medical people. But I have been in other medical facilities and hospitals and, I think this is where I want to be’

### Accessibility

Some participants spoke of their unique privilege in being able to access personalized medical services for complex health crises at the Alex Seniors Clinic. One man recounted an incident where the physician from the clinic gave him a home-visit.

‘I said, ‘I am still in a muddle, I don’t know what’s going on’, and 15 minutes later, there was a doctor at my door. You tell me where else that would happen?’

Others, however, expressed being negatively impacted by the perceived neglect of clinic staff.

‘I was not so disappointing but sometimes overlooking me. It is too busy sometimes here. I can tell them that I want to reach here, ‘Dr. X will reach you back’, and they do sometimes but sometimes they forget to do it and I think maybe they are too busy but I as a patient, sometimes disheartened.’

### Respect and support

Participants expressed great appreciation for being respected for their autonomous choices by physicians at the Alex Seniors Clinic, even when those choices are not necessarily healthy.

‘well, I don’t tell her any lies, it makes me feel good that I can just talk to her so openly’.

‘I should say no smoking but I can’t. We both smoke. Like Dr. M said, after all we’ve been through, she won’t insist that we quit. I know it’s not healthy but what the heck?’

### Advocacy

When participants lost their ability to be fully independent, they reported that clinic staff advocates on their behalf to ensure that they are receiving needed assistance. One participant detailed his appreciation for his physician who maintained regular contact with him and ensured he was receiving good home care.

‘She has gone out of her way to write letters to home care to make sure that I am being taken care of. Not every doctor would do that, I don’t think. Or, she has made appointments for me to come in, to make me feel more at ease. If it weren’t for her, I probably would be dead…’

## Discussion

The aim of this study was to explore patients’ perceptions of the factors impacting their health needs as well as the Alex Seniors Clinic’s responsiveness to these needs. The results indicate that participants perceive environmental, social, and personal factors as impacting their health. These findings are consistent with the literature reporting that housing, income security, safety, social contact and networks, health care, transportation, autonomous decision-making, and sense of self-worth [21,22] are key determinants of seniors’ health in Canada.

The nature and accessibility of care delivery, the respectful and supportive patient approach, and the advocacy for patients in the community were all ways that participants perceived the clinic as attempting to address their health needs. Hence participants’ health needs were not strictly medical but rather concerned with the nature of care delivery. These findings fit with the mandate of the Alex Seniors Clinic, which is to provide patient-centered multisystem care that addresses numerous community and social factors. The significance of our findings is that the care aims of the clinic are congruent with participant perceptions of need, and are seen to be so by patients themselves. This is especially apparent in the advocacy domain. The appreciation expressed by participants regarding advocacy at the clinic might be indicative of a service feature provided by the Alex Seniors’ Clinic that is not universally experienced by patients elsewhere. Research on community health clinic involvement in socioeconomically vulnerable communities has demonstrated a commonly unmet need for community advocacy [23]. The importance of a respectful, trusting and supportive relationship with care providers has also been documented as allowing patients to feel that they can tell their stories, leading to a more active self-management patient role and contributing to treatment planning that is reflective of specific care needs [24].

While the clinic appears to perform particularly well in relation to advocacy and respect, we identified evidence that their access health needs are less successfully addressed.

Lack of access to health and community services has been identified as a significant problem for socioeconomically challenged patients, reflecting the importance of the Alex Senior Clinic’s mandate to increase access [23]. Participants were less universally satisfied with the Alex’s attempts to improve access to its services and identified some areas of care that require further action. These include improving outreach to socially isolated seniors. It is not known, from this study, whether clinic staff are aware of these perceived limitations of clinic services. It is likely that they are, but that expectations within the community about improved access may be difficult to fulfill since they reflect broader environmental and structural imperatives, rather than social or behavioral ones concerning personal interactions between clinic staff and its patients. Consequently, this finding demonstrates that improved community connections and expansion of services are issues that constantly need to be addressed. Without involving patients in service evaluation processes, it may be difficult to gain an understanding of where such perceived gaps in services lie and how to best continue to address these. To ensure that clinic endeavours align with community-perceived needs, clinics that serve vulnerable populations must continue to solicit active participation of community members in service planning, delivery, and evaluation, in keeping with Canadian Association of Community Health Centres (CACHC) principles.

The features of this type of primary care organization can be used as a model for holistic healthcare delivery beyond the Alex and the city of Calgary. Vulnerable populations require a unique healthcare approach. By understanding the key factors affecting patient health needs from both the patient- and agency-perspective, clinics can begin to expand their scope according to patient needs.

## Conclusions

Participants indicated that their environmental, social and personal health needs were being addressed by the Alex Seniors Clinic through strategies related to accessibility, a respectful and supportive relationship as well as community advocacy. Apparent from the findings is that while respect and support, as well as advocacy, were effectively addressing needs, participants felt that accessibility problems continue to be health-related barriers for clinic patients. This may be due to the fact that issues of accessibility reflect larger community and social problems. Nevertheless, it is only through engaging the patient community for input on clinic approaches, that an understanding can be gained of how closely a clinic’s care goals are currently aligned with patient perspectives of the care and services they receive.
